# Native whey protein with high levels of leucine results in similar post-exercise muscular anabolic responses as regular whey protein: a randomized controlled trial

**DOI:** 10.1186/s12970-017-0202-y

**Published:** 2017-11-21

**Authors:** Håvard Hamarsland, Anne Lene Nordengen, Sigve Nyvik Aas, Kristin Holte, Ina Garthe, Gøran Paulsen, Matthew Cotter, Elisabet Børsheim, Haakon B. Benestad, Truls Raastad

**Affiliations:** 1 Department of Physical Performance, Norwegian School of Sport Sciences, P.O. Box 4014 Ullevål Stadion, 0806 Oslo, Norway; 2 Department of Nutrition, Institute of Basic Medical Sciences, University of Oslo, P.O. Box 1046, Blindern, 0317 Oslo, Norway; 3 Norwegian Olympic Federation, Oslo, Norway; 4 Arkansas Children’s Nutrition Center, Little Rock, AR USA; 5 Arkansas Children’s Research Institute, Little Rock, AR USA; 6 Departments of Pediatrics and Geriatrics, University of Arkansas for Medical Sciences, Little Rock, AR USA; 7 Section of Anatomy, Institute of Basis Medical Sciences, University of Oslo, Oslo, Norway

**Keywords:** Skeletal muscle, Supplementation, Amino acids, Protein quality, Stable isotopes, Resistance training, Nutrition

## Abstract

**Background:**

Protein intake is essential to maximally stimulate muscle protein synthesis, and the amino acid leucine seems to possess a superior effect on muscle protein synthesis compared to other amino acids. Native whey has higher leucine content and thus a potentially greater anabolic effect on muscle than regular whey (WPC-80). This study compared the acute anabolic effects of ingesting 2 × 20 g of native whey protein, WPC-80 or milk protein after a resistance exercise session.

**Methods:**

*A total of* 24 young resistance trained men and women took part in this double blind, randomized, partial crossover, controlled study. Participants received either WPC-80 and native whey (*n* = 10), in a crossover design, or milk (*n* = 12). Supplements were ingested immediately (20 g) and two hours after (20 g) a bout of heavy-load lower body resistance exercise. Blood samples and muscle biopsies were collected to measure plasma concentrations of amino acids by gas-chromatography mass spectrometry, muscle phosphorylation of p70S6K, 4E–BP1 and eEF-2 by immunoblotting, and mixed muscle protein synthesis by use of [^2^H_5_]phenylalanine-infusion, gas-chromatography mass spectrometry and isotope-ratio mass spectrometry. Being the main comparison, differences between native whey and WPC-80 were analysed by a one-way ANOVA and comparisons between the whey supplements and milk were analysed by a two-way ANOVA.

**Results:**

Native whey increased blood leucine concentrations more than WPC-80 and milk (*P* < 0.05). Native whey ingestion induced a greater phosphorylation of p70S6K than milk 180 min after exercise (*P* = 0.03). Muscle protein synthesis rates increased 1–3 h hours after exercise with WPC-80 (0.119%), and 1–5 h after exercise with native whey (0.112%). Muscle protein synthesis rates were higher 1–5 h after exercise with native whey than with milk (0.112% vs. 0.064, *P* = 0.023).

**Conclusions:**

Despite higher-magnitude increases in blood leucine concentrations with native whey, it was not superior to WPC-80 concerning effect on muscle protein synthesis and phosphorylation of p70S6K during a 5-h post-exercise period. Native whey increased phosphorylation of p70S6K and muscle protein synthesis rates to a greater extent than milk during the 5-h post exercise period.

**Trial registration:**

This study was retrospectively registered at clinicaltrials.gov as NCT02968888.

**Electronic supplementary material:**

The online version of this article (10.1186/s12970-017-0202-y) contains supplementary material, which is available to authorized users.

## Introduction

Protein ingestion produces a strong anabolic stimulus that elevates muscle protein synthesis [34]. The ability of a serving of protein to stimulate muscle protein synthesis (MPS) is dependent on absorption and blood kinetics of amino acids [8, 28, 36, 40], amount of protein ingested [22, 42], and the amino acid composition of the protein source [3]. Only the essential amino acids (EAA), especially leucine, initiate an immediate increase in MPS [4, 39]. Being a rapidly digested protein with a high leucine content, whey has been shown to stimulate MPS more than equal amounts of casein and soy in the first hours after exercise [36]. Other studies found that despite differences in absorption kinetics and amino acid composition, when measured over a sufficient time interval (4–6 h), milk and casein appear to elevate MPS equally effective as whey protein [21, 33]. This may be so because both the amount of protein and the complete EAA profile is important for maintenance of MPS over time [10]. The mechanisms behind the anabolic effects of amino acids still remain to be fully elucidated. At the molecular level the mechanistic target of rapamycin complex 1 (mTORC1) and its substrates (p70S6K and 4E–BP1) are believed to largely be responsible for the protein synthetic response to resistance exercise and protein intake [35], with resistance exercise potentiating the effect of protein ingestion [24, 42].

Native whey protein is produced by the filtration of unprocessed raw milk. This production method leaves proteins intact and gives native whey higher leucine content than the more common whey protein concentrate (WPC-80), which is a product of cheese production. Furthermore, it has been shown that intake of native whey induces greater leucine blood concentrations than WPC-80 [16]. Consequently, we hypothesized that native whey is a more potent stimulator of MPS than WPC-80. The aim of the current study was therefore to compare the time-dependent changes in mTORC1 substrate signalling and MPS in response to two 20 g doses of WPC-80 or native whey ingested immediately and two hours after leg resistance exercise. Moreover, we included a group consuming equal amounts of milk protein in order for a comparison against a commonly consumed source of protein in the Norwegian diet to be made [17]. To investigate whether potential differences in MPS between supplements had an effect on recovery we measured force-generating capacity before, 10 min after, 300 min after and 24 h after the workout.

## Materials and methods

### Participants and ethical approval

**Fig. 1:**
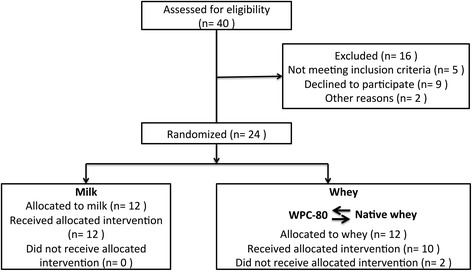
Participant flowchart

A total of 24 young men and women were included in the study (Table 1). Two participants withdrew from the study, due to busy time schedules. Results presented are from the remaining 22 participants (Fig. 1). All participants underwent a medical screening before entering the study. To take part participants had to be healthy and without any injuries to the musculoskeletal system that could interfere with the execution of training. Individuals with lactose intolerance, milk allergy or using any dietary supplements were excluded. Participants were strength trained (minimum one leg strength training session per week for the last six months) sport science students. The study was approved by the Regional Ethics Committee for Medical and Health Research of South-East Norway (2014/834/REK sør-øst C) and performed in accordance with the *Declaration of Helsinki*. All participants signed a written informed consent form before entering the study. The trial was registered at clinicaltrials.gov as NCT02968888.

**Table 1:** Participant characteristics

Characteristics	Milk	Whey	*P* values for group differences
N (♂/♀)	(8/4)	(5/5)	
Age (years)	25 ± 5	25 ± 2	0.575
Body mass (kg)	72.8 ± 12.4	70.0 ± 11.6	0.595
Lean body mass (kg)	57.1 ± 13.5	52.9 ± 9.6	0.426
Body fat (%)	19.1 ± 7.2	21.5 ± 6.4	0.407
Leg press 8 RM (kg)	210 ± 48	200 ± 53	0.662
Knee extensions 8 RM (kg)	87.1 ± 26.2	77.5 ± 17.3	0.335
Total weight lifted (kg)	9287 ± 2286	8766 ± 2170 / 8766 ± 2186	0.592

Characteristics of participants in the milk group, being exposed to the study once, and the whey group being exposed to the study two times, one with WPC-80 and one with native whey. Data shown as mean ± SD

### Study design

**Fig. 2:**
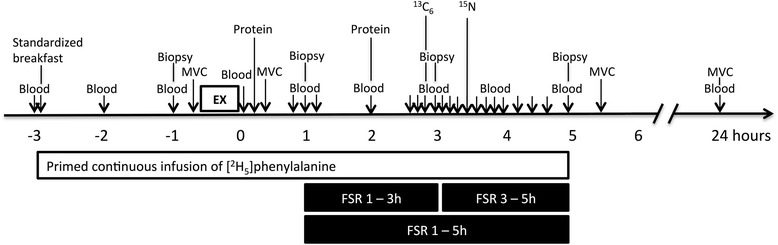
Experimental design

This study was a double blinded, partial crossover, randomized control trial (Fig. 2). Each participant was assigned to one of two groups. The randomization was stratified based on lean body mass. The milk group did the protocol once, whereas the whey group was exposed to the protocol two times, once consuming WPC-80 and once consuming native whey, in a randomized order, approximately two weeks apart. 3 h after a standardized breakfast participants performed an intense bout of high-load leg-resistance exercise. 20 g of protein from milk, WPC-80 or native whey, was ingested both immediately after, and again 2 h after exercise. Blood samples were collected from an antecubital vein to measure changes in blood concentrations of amino acids, glucose, insulin, urea and creatine kinase (CK). MPS and related intracellular signalling were measured during a 5-h recovery period combining biopsies and tracer infusion of [^2^H_5_]phenylalanine. In addition, we measured recovery of muscle force-generating capacity by maximal isometric voluntary contractions (MVC) for 24 h after exercise.

### Familiarization

During the two weeks prior to the study, participants met twice in the lab to establish their 8 repetition maximum (RM) in bilateral leg press and knee extension, and to perform familiarization to the standardized workout and the MVC-test. All participants were asked to refrain from physical exercise for 48 h prior to the experiments.

### Diet

At each familiarization session participants completed a 24-h dietary recall interview. A trained dietician conducted the recall interviews and analysed dietary nutrient content using the software Mat på Data 5.1 (Mattilsynet, Oslo, Norway, 2009). To standardize the diet participants were provided with a diet plan and pre-packaged food for the day before the experiment, and for the rest of the experimental period (2.5 days in total). The diet plan was individualized relative to body mass and provided participants with 40 kcal/kg and 1.5 g protein/kg per day. The standardized breakfast contained 23 kJ, 0,11 g protein, 0,30 g fat and 0.58 g carbohydrates per kg body mass.

### Infusion and exercise protocol

Participants arrived in the lab after an overnight fast. A cannula was inserted into a forearm vein in both arms. A baseline blood sample was drawn before participants ingested a standardized breakfast. The breakfast, containing 0.14 g of protein • kg body mass^−1^, was to be consumed within 20 min. Thirty minutes after the baseline blood sample a primed continuous infusion of [^2^H_5_]phenylalanine (0.05 μmol·kg^−1^·min^−1^; 2 μmol·kg^−1^ prime; Cambridge Isotopes Laboratories, Andover, MA, USA) was started. In addition to the constant infusion of [^2^H_5_]phenylalanine a bolus of [^13^C_6_]phenylalanine and [15 N]phenylalanine, not related to the results of this study, were infused at 170 and 200 min, respectively. Biopsies and blood samples were collected according to Fig. 2. The exercise session consisted of 4 sets of 8 repetitions to failure (8 RM sets) of leg press and knee extension, with a new set starting every 3 min. Warm-up sets of 10 repetitions at 50% and 80% of the 8RM loads were carried out in leg press.

### Supplements

Tine ASA (Oslo, Norway) produced the milk and whey supplements for this study. In order to match all drinks on macronutrients, cream (Tine, Norway), lactose (Arla food ingredients, Denmark), and water was added to WPC-80 and native whey (Table 2). Native whey and WPC-80 contained whey protein only, whereas milk contained 20% whey and 80% casein. Drinks were enriched with 6% [^2^H_5_] phenylalanine in order to maintain the plasma enrichment after intake. All drinks were matched for appearance and flavour.

**Table 2:** Amino acid and macronutrient content in supplements

	Amino acids (per serving)		
	Native whey	WPC-80	Milk
Alanine	1.1	1.0	0.6
Arginine	0.6	0.5	0.7
Aspartic acid	2.5	2.2	1.6
Cysteine	0.6	0.4	0.2
Phenylalanine	0.8	0.7	1.0
Glutamic acid	3.8	3.6	4.3
Glycine	0.4	0.4	0.4
Histidine	0.4	0.4	0.6
Isoleucine	1.2	1.3	1.0
Leucine	2.7	2.2	2.0
Lysine	2.3	1.9	1.7
Methionine	0.5	0.4	0.5
Proline	1.1	1.3	2.0
Serine	1.0	1.1	1.1
Threonine	1.1	1.5	0.9
Tyrosine	0.6	0.4	0.8
Valine	1.1	1.2	1.3
Tryptophan	0.5	0.3	0.3
Total protein	21.2	19.7	20.5
Fat	6.9	6.7	6.3
Carbohydrate	40.7	42.0	38.2

### Dual-energy X-ray absorptiometry

Body composition was assessed by dual energy X-ray absorptiometry (Lunar iDXA GE Healtcare, Madison, Wisconsin, USA, using the enCORE Software Version 14.10.022). After an overnight fast, before one of the familiarization sessions, participants were scanned from head to toe in a supine position, providing values for lean tissue, fat mass and bone mineral content. The coefficient of variation (CV) for the assessment of lean tissue was <1.1%.

### Maximal strength

Unilateral maximal knee extension strength was assessed in an isometric voluntary maximal contraction (MVC) in a custom-made knee-extension apparatus (Gym2000, Geithus, Norway). Participants were seated in a chair with a four-point belt fixing the chest and hips, with 90° in the hip and knee joints. Three attempts of 5 s with 1 min rest between were given to reach MVC force. Force was measured with a force transducer (HMB U2 AC2, Darmstadt, Germany). MVC was tested after a 5 min warm up on a cycle ergometer, except for 10 min after the workout. MVC was tested unilaterally and no effect of the biopsy procedure was observed.

### Blood analyses

Blood serum was obtained by clotting for 30 min at room temperature before centrifugation. Plasma was obtained by immediate centrifugation in lithium heparin tubes, respectively. Both serum and plasma were centrifuged at 4 °C for 10 min at 1300 g, and stored at −80 °C until analysis. Serum samples were analyzed for creatine kinase (CV: 2.8%) and urea (2.2%) at Fürst Medical Laboratory (Oslo, Norway). Plasma samples were analyzed for concentrations of insulin (6.8%) using enzyme-linked immune sorbent assay (Alpco, Salem, NH, USA), and glucose (2.1%) using a Cobas clinical analyzer (Cobas 6000, Roche Diagnostics, Indianapolis, IN, USA). Amino acid concentrations were measured in plasma as described earlier [16] with a EZfaast amino acid analysis kit (Phenomenex®, Torrance, CA, USA) and gas chromatography/mass spectrometry (Shimadzu QP-2010 Ultra GCMS, Shimadzu Scientific Instruments, Columbia, MD).

### Biopsy collection and pre-analytical processing

Muscle biopsies were collected from the mid portion of *m. vastus lateralis* with a modified Bergström technique with suction. Pre-analytical processing of muscle tissue was performed as per Paulsen and colleagues [26]. In short specimens were used to make a homogenate of soluble protein for Western blotting analyses and for analysis of MPS.

### Western blot

Samples for Western blot were treated as previously described [26], quantified with ChemiDoc MP (BioRad Laboratories, CA, USA) and analyzed with Image Lab (v5.1, BioRad Laboratories, CA, USA). All samples were run in duplicate. Antibodies against p70S6K and phosphor-p70S6K Thr^389^, Eukaryotic elongation factor 2 (eEF-2), phospho-eEF-2^Thr56^, Eukaryotic initiation factor 4E–binding protein 1 (4EBP-1), phospho-4EBP-1^Thr37/46^, and secondary anti-rabbit were purchased from Cell Signalling Technology (Beverly, MA, USA).

### Blood, muscle protein-bound and intracellular free phenylalanine enrichment

Plasma from blood samples for the measurement of phenylalanine enrichment was analysed as previously described [43]. Briefly, plasma was deproteinised with 500 μl 15% sulfosalicylic acid, and amino acids were purified using cation exchange chromatography (AG 50 W-8X, 100–200 mesh H+ form; Bio-Rad Laboratories, Richmond, CA, USA). Purified amino acids were dried under vacuum (Vacuum Dry Evaporator System, Labconco, Kansas City, MO, USA) and thereafter derivatised with 80 μl (1:1, *v*/v) acetonitrile: *N*-tert-butyldimethylsilyl-*N*-methyltrifluoroacetamide (MTBSTFA, Sigma-Aldrich) for 45 min at 100 °C. Isotopic enrichments of the plasma samples were determined on the tert-butyl dimethylsilyl (TBDMS) derivatives using gas chromatography/mass spectrometry (Shimadzu QP-2010 Ultra GCMS, Shimadzu Scientific Instruments, Columbia, MD, USA) and selected ion monitoring [43]. Enrichments were expressed as tracer-to-tracee ratios. Appropriate corrections were made for overlapping spectra [43].

Plasma from blood samples for the measurement of phenylalanine enrichment was analyzed as previously described [7]. Briefly, plasma was deproteinized by adding 20% perchloric acid and centrifuged at 1000×g at 4C for 10 min. Supernatant was removed, and the mixed plasma protein pellet was washed three times with 2% PCA, two times with ethanol, and one time with diethyl ether, dried overnight at 50 °C and hydrolysed overnight in 6 N HCl at 110 °C. The hydrolysed mixed plasma protein samples were then processed using the same method as muscle protein bound samples.

Twenty-five to thirty mg of muscle was placed in 800 μl 10% perchloric acid (PCA), homogenized and centrifuged. The supernatant was collected for measurement of intracellular amino acid enrichment. The remaining pellet (bound protein) was washed three times with 2% PCA, two times with ethanol, and one time with diethyl ether, dried overnight at 50 °C and hydrolysed overnight in 6 N HCl at 110 °C. Amino acids from the bound and intracellular fractions were then purified by cation exchange chromatography and thereafter derivatised in the same way as for the blood samples. Isotopic enrichment of TBDMS-phenylalanine in the bound protein was determined by gas chromatography-combustion-isotope ratio/mass spectrometry (GC-C-IRMS) set to high temperature conversion (HTC) mode for analysis of deuterium (Delta V Advantage Isotope Ratio Mass Spectrometer with GC Isolink, Thermo Scientific, West Palm Beach, FL, USA). Enrichment is calculated from the relative ratios of hydrogen (mass 3/mass 2) corrected for natural abundance of deuterium, divided by fraction of atoms that could be labelled (5/39).

### Calculations

Baseline muscle fractional synthesis rate (FSR) was calculated using the precursor product method [43]:

FSR (%h^−1^) = E_p2_ – E_p1_ / (E_pre_ x *t*) × 100.

The product is the difference in enrichment of the bound protein pool (E_p2_) and the mixed plasma proteins (E_p1_). The precursor (E_pre_) is the average plasma free or muscle free D_5_ phenylalanine enrichments to estimate the upper (muscle free) and lower (plasma free) limits of the true muscle protein FSR. The tracer incorporation time is denoted by *t*.

Skeletal muscle fractional synthesis rate (FSR) was calculated (as a measure of MPS) according to the precursor product method where the precursor is the mean enrichment of the intracellular pool (E_IC_) of biopsies being analysed [43]. The product is the difference in enrichment of the bound protein (E_BP_) pools of the two muscle biopsies being analysed. Skeletal muscle FSR is expressed as per cent per hour: FSR (%/hour) = ((E_BP*t*2_-E_BP*t*1_)/(E_IC_·(*t*_2_-*t*_1_)))·100.

The baseline MPS was only calculated during the first experiment for participants in the whey group, and this value was used as a baseline for both supplements in this group.

### Statistics

Non-normally distributed data (D’Agostino and Pearson omnibus normality test) were log-transformed prior to statistical analysis. All data are illustrated in original form. As the main goal of the study was the comparison of WPC-80 with native whey, these data were analysed by one-way repeated measures ANOVA. Comparisons against milk were done with a two-way ANOVA with repeated measures (time x group), with group as non-repeated and time as repeated. A Tukey’s and Dunnett’s test was used as post hoc tests to specify the significant differences between trials and time points (within groups), respectively. Subject characteristics and AUC responses between groups were analysed with a one-way non-repeated measure ANOVA. A sample size calculation was conducted using a power of 80% based on mixed muscle FSR results from an earlier study comparing whey and casein in young men [36] StatMate, Graphpad Software, San Diego, CA, USA). Based on the power calculation the goal was to include 10 subjects in each group. Statistical analyses were made using Prism Software (Graphpad 6, San Diego, CA, USA), All results are expressed as means ± standard deviation (SD). Statistical significance level was set at *p* ≤ .05.

## Results

Participant characteristics were not significantly different between groups (Table 1).

### Diet standardization and exercise variables

There were no differences between the groups in terms of caloric (milk: 42 ± 7 kcal·kg^−1^·day^−1^, WPC-80 and native whey: 40 ± 9 kcal·kg^−1^·day^−1^, *P* = 0.65) and protein intake (milk: 2.0 ± 0.5 g·kg^−1^·day^−1^, whey: 1.8 ± 0.3 g·kg^−1^·day^−1^, *P* = 0.12).

There were no differences between groups for total training volume (Table 1), 8RM in leg press (*p* = 0.66), knee extensions (*p* = 0.34), training volume (load x repetitions; *p* = 0.59) and rest intervals (p = 0.59–0.88) during the workout.

### Blood amino acid concentrations

**Fig. 3:**
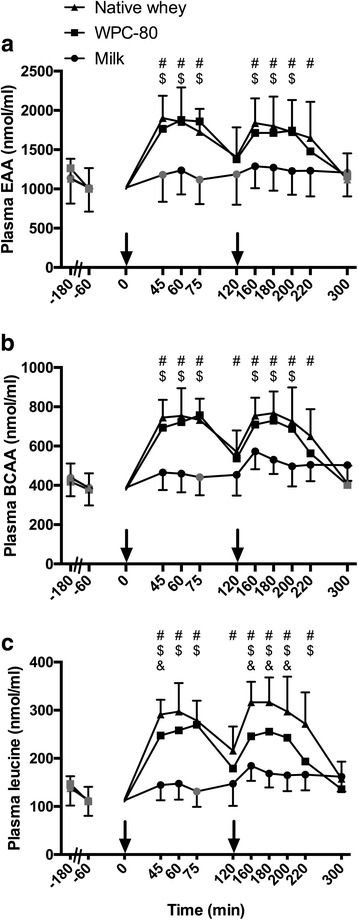
Blood concentrations of essential amino acids (**a**), branched chain amino acids (**b**) and leucine (**c**) following intake of milk, WPC-80 or native whey immediately and two hours after a bout of heavy leg resistance exercise. Arrows indicate time points of protein supplement ingestion. Values are mean ± SD (only shown for highest and lowest values). *n* = 12 in the milk group and 10 in the WPC-80 and native whey group. Black symbols are significantly different from resting values. # native whey greater than milk at the same time point; $ WPC-80 greater than milk at the corresponding time point; & native whey greater than WPC-80 at the corresponding point, *p* < 0.05

Ingestion of WPC-80 and native whey led to a robust increase in blood concentrations of total amino acids, EAA, total BCAA and individual BCAAs at nearly all time points, except 300 min (*P* < 0.001 for all, except valine: *P* < 0.03; Fig. 3). Native whey induced a higher concentration of leucine in blood than WPC-80 after both servings (*P* < 0.05). Milk ingestion also increased blood concentrations of total amino acids, EAA, total BCAA and individual BCAAs at most time points, but to a lesser extent than WPC-80 and native whey. Area under the curve for EAA, BCAA, isoleucine and leucine was 100–200% greater for both native whey and WPC-80 compared to milk (P < 0.001, data not shown). Native whey intake resulted in a greater area under the curve than WPC-80 (40%, P < 0.001) and milk (240%, P < 0.001) for leucine, and relative to milk (75%, *P* = 0.002) for total amino acids. Blood concentrations of individual EAA and non-essential amino acids are shown in Additional file 1: Figure S1 and Additional file 2: Figure S2, respectively.

### Blood glucose, insulin, urea and creatine kinase

**Fig. 4:**
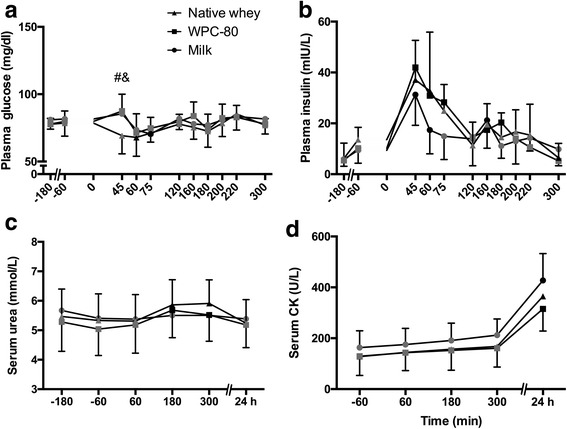
Blood concentrations of glucose (**a**), insulin (**b**), urea (**c**) and creatine kinase (**d**) following intake of 20 g milk protein, WPC-80 or native whey immediately after a bout of heavy leg resistance exercise. Arrows indicates time point of protein supplement ingestion. Values are mean ± SD (only shown for highest and lowest values). n = 12 in the milk group and 10 in the WPC-80 and native whey group. Black symbols are significantly different from resting values. # native whey greater than milk at the same time point; & native whey greater than WPC-80 at the same time point, p < 0.05

Plasma glucose remained relatively stable during the experiment for all groups. At 45 min native whey had significantly lower blood glucose than milk and WPC-80 (*P* > 0.05, Fig. 4a). Plasma insulin concentration increased in all groups after the first serving of supplements (60–300%) and returned to baseline before the second serving (Fig. 4b). Milk and WPC-80 increased plasma levels of insulin after the second serving (60–130%). Serum urea was increased 5–10% at 180 and 300 min after WPC-80 and native whey (*P* < 0.005; Fig. 4c). The area under the curve for urea was not significantly different between groups (*P* > 0.84). All groups increased blood levels of CK at 180 min (≈20%), 300 min (≈40%) and 24 h (≈200%; Fig. 4d). No group differences were observed for concentrations of insulin, urea and CK.

### Signalling

**Fig. 5:**
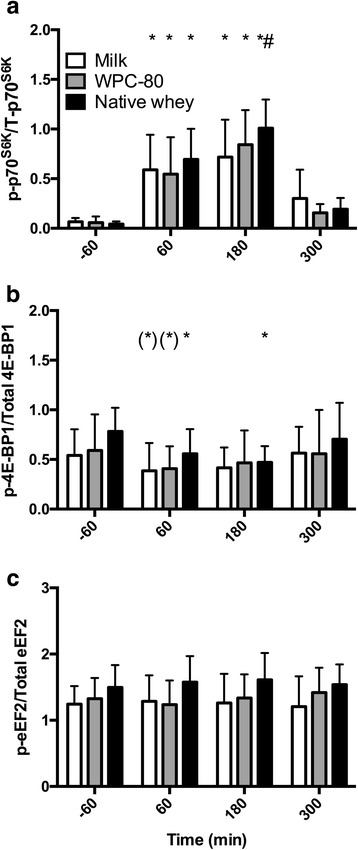
Ratio between phosphorylated and total p70S6K (**a**), 4E–BP1 (**b**) and eEF-2 (**c**) following intake of milk, WPC-80 or native whey immediately and two hours after a bout of heavy leg resistance exercise. Values are mean ± SD. n = 12 in the milk group and 10 in the WPC-80 and native whey group. * Different from resting values. # different from milk at the corresponding time point, p < 0.05

*p70S6K* phosphorylation increased in all groups at 60 and 180 min post-exercise (*P* < 0.001; see Fig. 5a). There were no differences between WPC-80 and native whey. At 180 min native whey showed a higher phosphorylation of p7S6K than milk (*P* = 0.03). Phosphorylation status of 4EBP-1 decreased at 60 (*P* = 0.018) and 180 (P < 0.001) min with native whey, and tended to decrease with milk and WPC-80 at 60 min after exercise (milk: *P* = 0.099, WPC-80: *P* = 0.066). There were no differences in phosphorylation status of 4EBP-1 between groups (Fig. 5b). Phosphorylation status of eEF-2 did not change, and there were no apparent differences between groups (Fig. 5c). Representative blots are shown in Additional file 3: Figure S3.

### Muscle protein synthesis

**Fig. 6:**
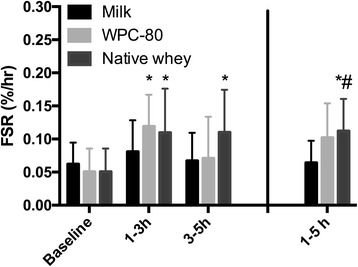
Mixed muscle FSR values following intake of milk, WPC-80 or native whey immediately and two hours after a bout of heavy leg resistance exercise. Values are mean ± SD. *n* = 10 in the milk group and 10 in the WPC-80 and native whey groups. # Different from milk over the corresponding time period, p < 0.05

[^2^H_5_]phenylalanine TTR in blood did not differ significantly between groups, but was significantly increased at 175 and 180 min with milk and WPC-80, and 175 min with native whey (Additional file 4: Figure S4). During the early (1–3 h) period WPC-80 (*P* = 0.044) and native whey (*P* = 0.025) increased MPS compared to baseline (Fig. 6). In the late period (3–5 h), this difference was maintained for native whey (*P* = 0.049) but not WPC-80. No group differences were observed for the separate early and late period. During the entire post-exercise period (1–5 h) MPS was not significantly different between native whey and WPC-80 ingestion, but MPS was higher after native whey ingestion than after milk ingestion (*P* = 0.023).

### Recovery of muscle function

**Fig. 7:**
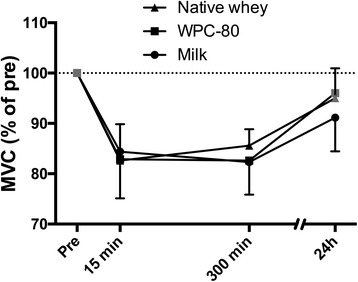
Isometric knee extensor force-generating capacity relative to resting values following intake of milk, WPC-80 or native whey immediately and two hours after a bout of heavy leg resistance exercise. Values are mean ± SD. Black symbols are significantly different from baseline, p < 0.05

A reduced force-generating capacity measured as MVC force, was evident 15 min after the exercise session in all groups (native whey: −17.4 ± 7.3%, WPC-80: -17.1 ± 7.4%, milk: −16.6 ± 9.3%, *P* < 0.001; Fig. 7). At 24 h WPC-80 and native whey were not significantly different from baseline (*P* > 0.098), whereas milk had a lower force generating capacity than baseline (P < 0.001). There were no significant group differences.

### Correlations

The area under the curve for blood leucine concentrations correlated with p70S6K phosphorylation at 60 and 180 min (*r* = 0.48 and 0.55, *P* < 0.05, data not shown).

## Discussion

The present study tested the hypothesis that native whey would have a greater acute anabolic effect on muscle than WPC-80, when supplemented as 20 g protein doses immediately and two hours after resistance exercise in young participants. Despite a larger increase in blood leucine concentrations, native whey stimulated post-exercise (1–5 h) p70S6K-phosphorylation and increased rates of MPS to a similar extent as WPC-80 after resistance exercise. However, compared to milk, native whey induced larger increases in blood concentrations of EAA and leucine, greater phosphorylation of p70S6K, and higher rates of MPS.

As observed in previous studies, blood amino acid concentrations reached higher levels at most time points for the individual BCAAs and EAA, after the whey supplements compared to milk ingestion [16, 21]. In support of the anabolic effect of leucine we observed a moderate correlation between area under the curve for blood concentrations of leucine and the phosphorylation of p70S6K. According to the leucine threshold hypothesis, a high blood or intracellular concentration of leucine is considered a prerequisite for maximal stimulation of MPS [29]. Changes in blood amino acid concentrations are not only due to differences in digestion and absorption rates. It is also dependent on the release of amino acids from muscle due to MPB and the ability of muscle to take up amino acids from blood. Unfortunately, we were not able to measure MPB or intracellular amino acids. Based on previous studies we can assume the intracellular changes in amino acid concentrations are less than those observed in blood [5, 14].

The time course of p70S6K phosphorylation followed the same pattern in all groups peaking at 180 min, before returning towards baseline at 300 min. This is in line with previous studies investigating the effect of protein supplementation after resistance exercise [2, 15, 32, 33]. On the group level, p70S6K phosphorylation results were consistent with the higher total MPS-rates with native whey compared to milk. However, on the individual level there were no correlations between p70S6K phosphorylation and MPS. This disassociation between blood EAA/leucine concentrations, signalling and MPS has previously been observed in similar studies [2, 14, 15].

We observed an overall decrease (only significant with native whey) in the phosphorylation of 4E–BP1 after resistance exercise and protein supplementation during the first hours after resistance exercise. Generally, the phosphorylation of 4E–BP1 is expected to increase after resistance exercise [12, 18, 23], but not all studies support this finding [1, 40]. Although, both p70S6K and 4E–BP1 are downstream of mTORC1, they do not respond similarly to rapamycin treatment. Thus, other mechanisms than mTORC1 activity are also affecting the phosphorylation state of these kinases, possibly leading to the observed differences [37]. In opposition to the observed lack of response to rapamycin, which is believed to imitate amino acid signalling [19], 4E–BP1 has been shown to respond to protein intake [23] and it is possible that 4E–BP1 phosphorylation already was elevated at baseline, due to the standardized breakfast in our study. We failed to observe any nutrient or exercise induced changes in eEF-2 phosphorylation, this have also been reported by others [1, 23].

We did not find a significant different MPS-response to ingestion of 20 g of WPC-80 or native whey, during the 5-h post exercise period. Both WPC-80 and native whey were likely able to maximally stimulate MPS with the applied supplementation regime [25, 42]. If supplemented as a suboptimal dose or in elderly, the higher leucine content of native whey may have resulted in a greater anabolic effect than WPC-80. However, previous studies showing an effect of added leucine on MPS have applied a substantially larger difference in the leucine content of supplements [3, 9]. Consequently, whether the moderate difference in leucine content between WPC-80 and native whey will have a meaningful effect in these settings is unclear. Native whey, in contrast to WPC-80, was able to maintain MPS-rates significantly higher than baseline during the late period. Although native whey was not significantly different from the other supplements during the late period, the observed pattern is interesting. The question as to whether the optimal feeding frequency is affected by protein type should be investigated in future studies.

Few studies have directly compared the acute anabolic responses to ingestion of milk and whey proteins. As milk protein is primarily composed of casein (80%) and a smaller part of whey (20%), some information might be gained from studies comparing these purified fractions. Such studies suggests there are only minor differences, if any, in MPS response between ingesting whey and casein after resistance exercise if measured over a sufficient amount of time, e.g. 4–6 h [33, 38]. However, there seems to be a temporal difference between the effects of whey and casein, with whey inducing a faster [36] more transient increase in MPS, whereas casein ingestion results in a slower more prolonged increase in MPS after resistance exercise [33]. We observed a similar pattern as that reported by Reitelseder and colleagues [33], with the whey supplements increasing MPS during the early post-exercise period (1–3 h), whereas milk did not. However, our second serving makes it difficult to compare the late periods between studies. As 18 g of milk protein previously have been shown to increase MPS in young men after resistance exercise [41] we expected our supplementation of 2 × 20 g of milk protein to elicit a measurable effect on MPS. Perhaps ingesting the milk supplement as a single serving of 40 g instead of 2 × 20 g would have elicited a greater effect. It is also possible that our standardized breakfast stimulated MPS at baseline and the change from the fasted state would have been greater.

In order to investigate the effects of slow and fast increases in blood amino acid concentrations, without the challenge of different amino acid content, West and colleagues [40] supplemented participants with either a bolus (25 g) or a pulse (10 × 2.5 g every 2 min; mimicking casein) of whey protein. Anabolic signalling and MPS was greater with the bolus during a 5-h post-exercise period, indicating that appearance of amino acids in blood affect the anabolic response. The milk, WPC-80 and native whey supplements in the current study all contained 2.0, 2.2 and 2.7 g of leucine per serving. Thus, all servings contained an amount of leucine above 1.8–2.0 g, which has been estimated to be needed in order to maximally stimulate MPS in young individuals [31]. Thus, leaving the varying digestion rate of milk and whey as the most likely explanation for our observed differences in anabolic response between milk and native whey.

Our resistance exercise protocol led to a 5–30% reductions in muscle force-generating capacity (10 min after exercise). These ranges of reductions in muscle function indicate mild to moderate muscular stress, which is supported by small increases in CK across groups [27]. In accordance with a previous study, no differences were observed in recovery of muscle function between groups [16]. The clear effect of protein on anabolic signalling and MPS may theoretically accelerate recovery of muscle function, as has been shown in studies applying more damaging eccentric muscle contractions [6, 11]. In the current study, a workout considered more “normal” and less muscle damaging was applied, while at the same time investigating more comparable supplements, making it less likely to observe a difference.

The lack of a direct measure of muscle protein breakdown (MPB) is a limitation in this study, as both MPS and MPB are needed to calculate net protein balance in muscle. Previous studies have shown MPS to respond with greater changes than MPB [5, 30] and MPB to be a minor determinant of net muscle protein balance in the acute response to protein intake following resistance exercise [13]. Thus, we assume that our MPS measurements to large extent reflect the major part of the net protein balance response. [^2^H_5_]phenylalanine TTR in blood was not affected by the supplements. The short-lived increase in [^2^H_5_]phenylalanine TTR was due to a bolus infusion of [^13^C_6_]phenylalanine not related to the results in this study. This increase would not affect the IRMS measures of bound protein but could potentially alter the GCMS measures of intracellular [^2^H_5_]phenylalanine. When analysed, intracellular enrichment of [^2^H_5_]phenylalanine were at steady state and did not reflect the brief fluctuations in blood. Measures of MPS should therefore not be affected.

## Conclusion

Native whey is a relatively new form of whey protein, which due to its high leucine content has received substantial attention and claims [20]. In this study we compared native whey with regular whey protein after a bout of resistance exercise. Despite a higher increase in blood leucine concentrations after ingestion of native whey, there were no differences between native whey and WPC-80 in stimulating the phosphorylation p70S6K, eEF-2 and 4E–BP1, MPS during a 5-h post exercise period. Native whey increased post-exercise phosphorylation of p70S6K more and had a higher rate of MPS than milk during the 5-h post exercise period. The higher rates of MPS with native whey compared to milk do not necessitate a greater adaptation over time. In order to investigate the potential differences in adaptation over time a long-term training study is needed.

## Additional files

Additional file 1: Figure S1. Blood concentrations of essential amino acids (except leucine) following intake of 20 g milk protein, WPC-80 and native whey after a bout of resistance exercise. Arrows indicate time points of protein supplement ingestion. Values are mean ± SD (only shown for highest and lowest values). *n* = 12 in the milk group and 10 in the WPC-80 and native whey group. Black symbols are significantly different from resting values. # native whey greater than milk at the same time point; $ WPC-80 greater than milk at the corresponding time point; & native whey greater than WPC-80 at the corresponding point, *p* < 0.05. (TIFF 989 kb)

Additional file 2: Figure S2. Blood concentrations of non-essential amino acids following intake of 20 g milk protein, WPC-80 and native whey after a bout of resistance exercise. Arrows indicate time points of protein supplement ingestion. Values are mean ± SD (only shown for highest and lowest values). n = 12 in the milk group and 10 in the WPC-80 and native whey group. Black symbols are significantly different from resting values. # native whey greater than milk at the same time point; $ WPC-80 greater than milk at the corresponding time point, p < 0.05. (TIFF 936 kb)

Additional file 3: Figure S3. Representative blots from Western Blot analysis of phosphorylated and total p70S6K, 4E–BP1 and eEF-2. Bands are shown for rest, 1, 3 and 5 h after exercise. Samples were run in duplicate. (TIFF 539 kb)

Additional file 4: Figure S4. [^2^H_5_]phenylalanine tracer to tracee ratio in plasma. Values are mean ± SD (only shown for highest and lowest values). *n* = 10 and 10 in the milk and native whey group, respectively. Filled symbols are significantly different from baseline, *p* < 0.05. (TIFF 190 kb)
