# Supplementary figures and images for: Native whey protein with high levels of leucine results in similar post-exercise muscular anabolic responses as regular whey protein: a randomized controlled trial
Source: J Int Soc Sports Nutr. 2017 Nov 21;14:43. doi: 10.1186/s12970-017-0202-y (PMC5697397; doi:10.1186/s12970-017-0202-y)

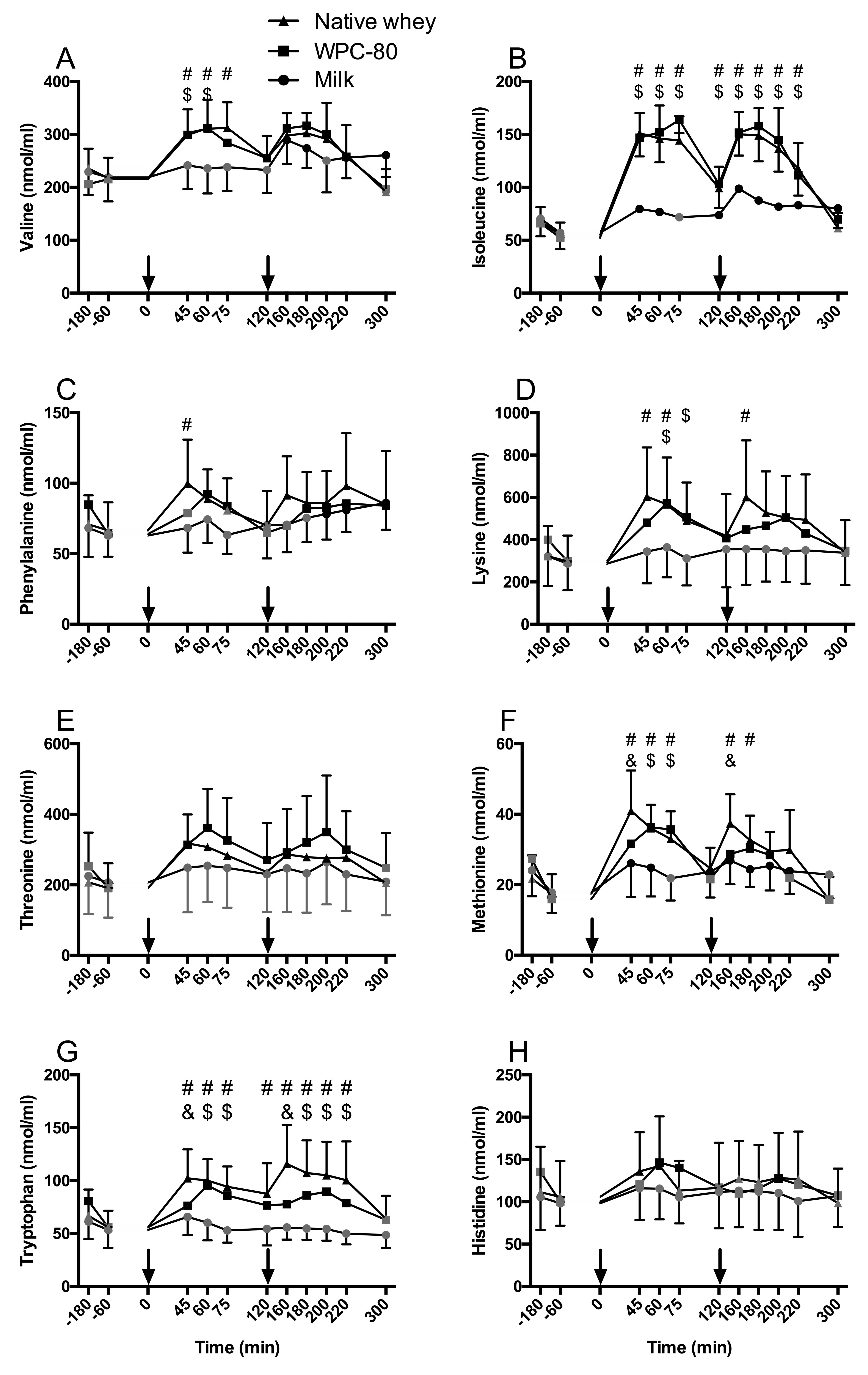

Supplement: Supplementary file 1 — Blood concentrations of essential amino acids (except leucine) following intake of 20 g milk protein, WPC-80 and native whey after a bout of resistance exercise. Arrows indicate time points of protein supplement ingestion. Values are mean ± SD (only shown for highest and lowest values). n = 12 in the milk group and 10 in the WPC-80 and native whey group. Black symbols are significantly different from resting values. # native whey greater than milk at the same time point; $ WPC-80 greater than milk at the corresponding time point; & native whey greater than WPC-80 at the corresponding point, p < 0.05. (TIFF 989 kb) [file 12970_2017_202_MOESM1_ESM.tiff]

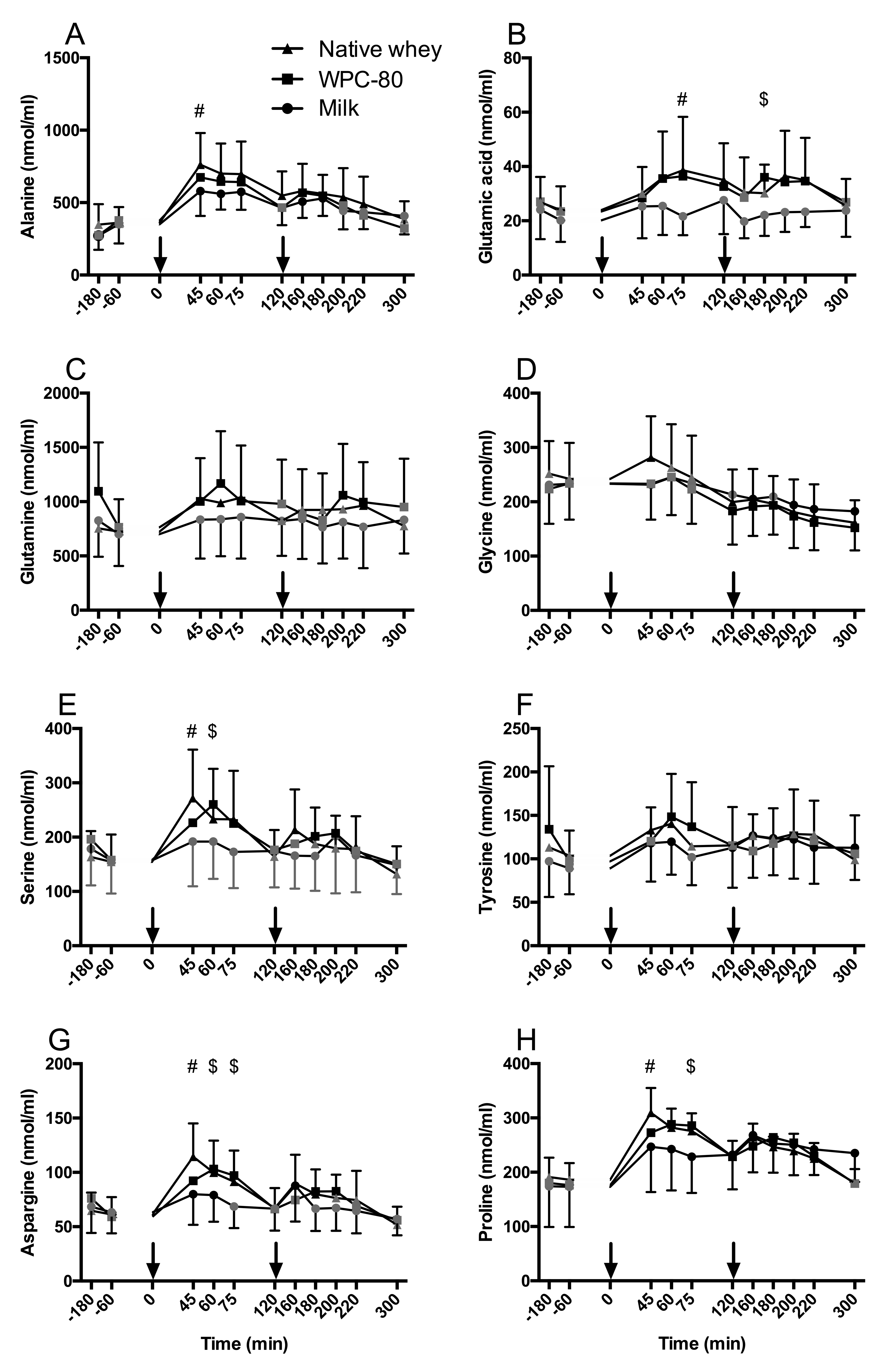

Supplement: Supplementary file 2 — Blood concentrations of non-essential amino acids following intake of 20 g milk protein, WPC-80 and native whey after a bout of resistance exercise. Arrows indicate time points of protein supplement ingestion. Values are mean ± SD (only shown for highest and lowest values). n = 12 in the milk group and 10 in the WPC-80 and native whey group. Black symbols are significantly different from resting values. # native whey greater than milk at the same time point; $ WPC-80 greater than milk at the corresponding time point, p < 0.05. (TIFF 936 kb) [file 12970_2017_202_MOESM2_ESM.tiff]

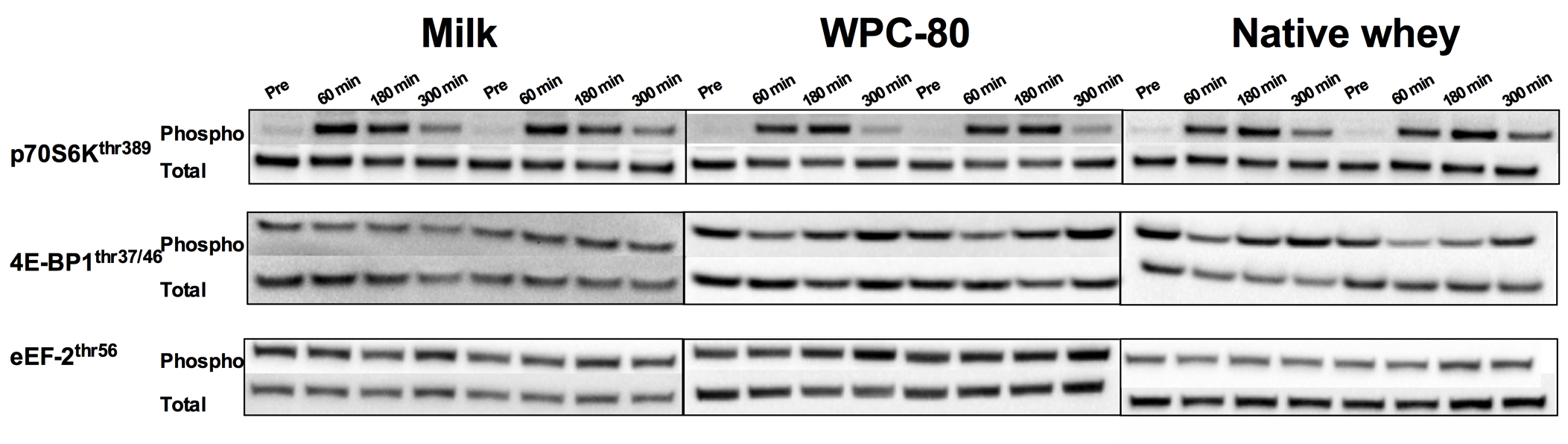

Supplement: Supplementary file 3 — Representative blots from Western Blot analysis of phosphorylated and total p70S6K, 4E–BP1 and eEF-2. Bands are shown for rest, 1, 3 and 5 h after exercise. Samples were run in duplicate. (TIFF 539 kb) [file 12970_2017_202_MOESM3_ESM.tiff]

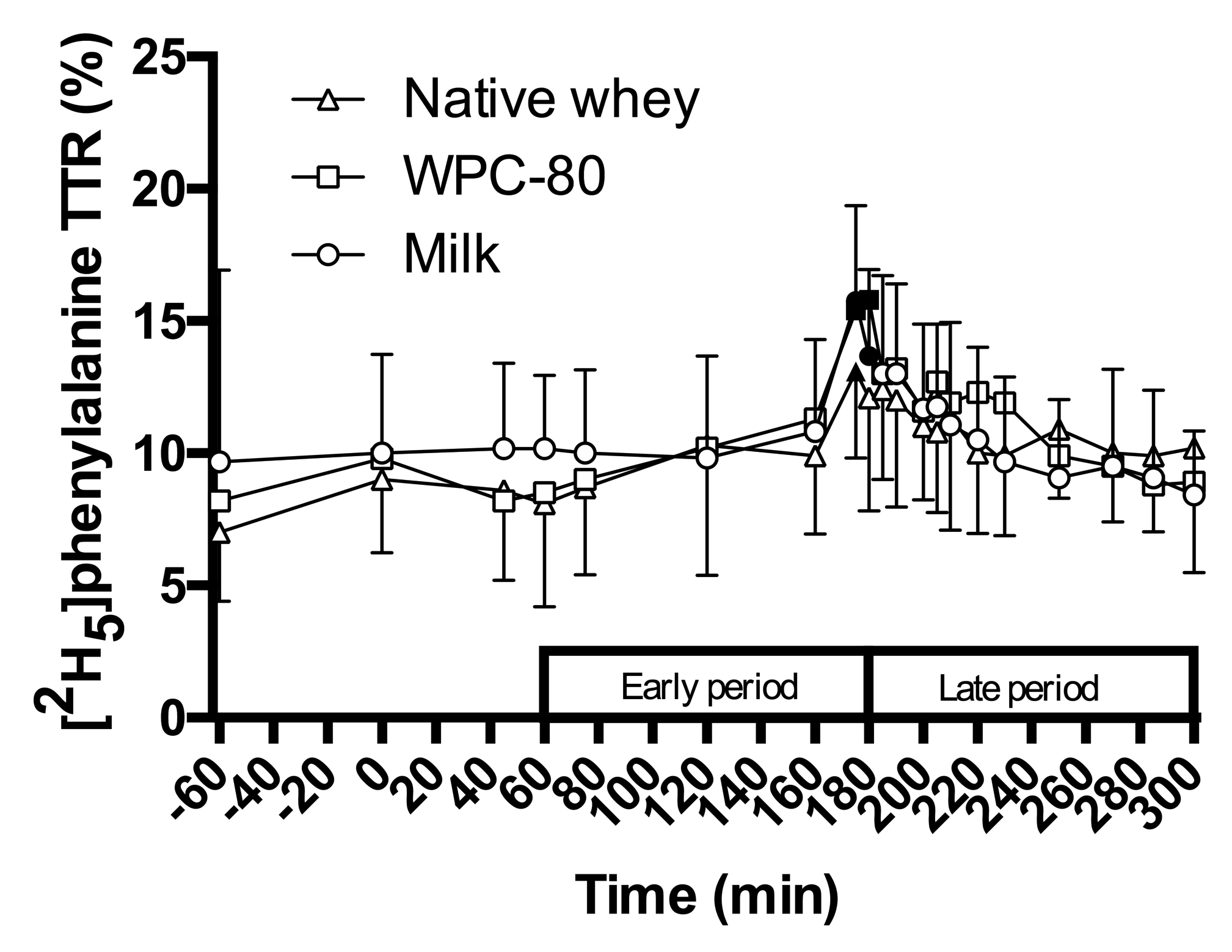

Supplement: Supplementary file 4 — [2H5]phenylalanine tracer to tracee ratio in plasma. Values are mean ± SD (only shown for highest and lowest values). n = 10 and 10 in the milk and native whey group, respectively. Filled symbols are significantly different from baseline, p < 0.05. (TIFF 190 kb) [file 12970_2017_202_MOESM4_ESM.tiff]
